# Supplementary material for: Effects of Phytochelatin-like Gene on the Resistance and Enrichment of Cd2+ in Tobacco
Source: Int J Mol Sci. 2022 Dec 18;23(24):16167. doi: 10.3390/ijms232416167 (PMC9784533; doi:10.3390/ijms232416167)
Supplement: Supplementary file 1 [file ijms-23-16167-s001.zip › ijms-2093198-supplementary.pdf]

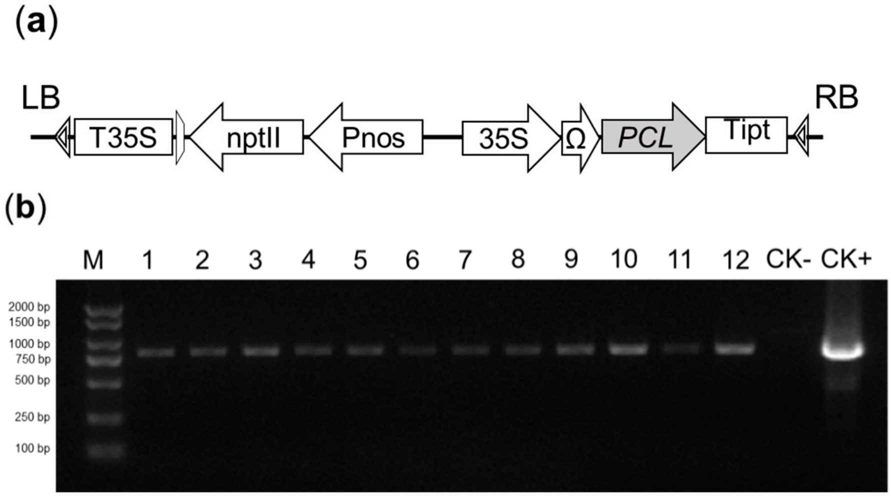

Figure S1. Molecular confirmation identification of 35S-PCL transgenic tobacco. **(a)** Schematic map of the vector used for tobacco transformation. (**b)** Detection of target genes in transgenic tobacco plants. LB: left border; RB: right border; t35S: CaMV 35S terminator; nptII: the neomycin phosphotransferase II gene; Pnos: nopaline synthase promoter; 35S: CaMV 35S promoter; Ω: Ω translational enhancer of the TMV; Tipt: terminator of the isopentyl transferase gene of Agrobacterium tumefaciens C58; M: DL 2000 Marker; 1-12: detection of target genes in transgenic tobacco plants; CK-: negative control with WT tobacco gDNA as a template; CK+: positive control with *35S-PCL* plasmid as template.

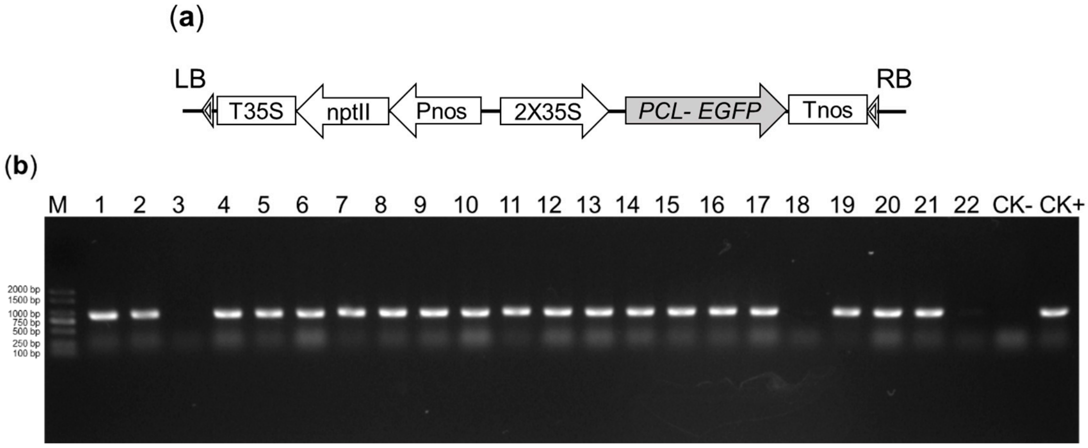

Figure S2. Molecular confirmation identification of p35S-PCL-eGFP transgenic tobacco. **(a)** Schematic map of the vector used for tobacco transformation. (**b)** Detection of target genes in transgenic tobacco plants. RB: right border; t35S: CaMV 35S terminator; nptII: the neomycin phosphotransferase II gene; Pnos: nopaline synthase promoter; 2X35S: the double CaMV 35S promoter; EGFP: enhanced green fluorescent protein; Tnos: nopaline synthase terminator; M: DL 2000 Marker; 1-22: detection of target genes in transgenic tobacco plants; CK-: negative control with WT tobacco gDNA as a template; CK+: positive control with *p35S-PCL-eGFP* plasmid as template.
